# Cardiovascular Catastrophe in Hysteroscopic Surgery: A Case of Diagnostic Dilemma, Arrest, and Recovery

**DOI:** 10.7759/cureus.98513

**Published:** 2025-12-05

**Authors:** Bheemas Atlapure, Veswudu Swuro, Anirban Bhattacharjee, Ankur Khandelwal, Himangshu Malakar, Saswati Tripathy, Satheesh G, Habib Md R Karim

**Affiliations:** 1 Anaesthesiology, Critical Care, and Pain Medicine, All India Institute of Medical Sciences, Guwahati, Guwahati, IND; 2 Obstetrics and Gynaecology, All India Institute of Medical Sciences, Guwahati, Guwahati, IND

**Keywords:** acute pulmonary edema, dilutional hyponatremia, fluid overload, hysteroscopic surgery, intraoperative cardiac arrest, operative hysteroscopic intravascular absorption syndrome

## Abstract

Hysteroscopic surgery carries a risk of fluid overload and electrolyte imbalance. Although acute cardiovascular collapse due to excessive fluid absorption is rare, it can be life-threatening. A 36-year-old otherwise healthy female underwent hysteroscopic myomectomy under combined spinal-epidural anesthesia. She developed sudden bradycardia, hypotension, and unresponsiveness 50 minutes into the procedure, which responded to adrenaline and brief chest compressions (<1 minute). She was intubated and achieved return of spontaneous circulation (ROSC); however, elevated airway pressures complicated the case. The hysteroscopic procedure was continued for another 20 minutes, considering near-completion, but required conversion to an open approach. Meanwhile, desaturation was noted, and the patient had pulseless ventricular tachycardia requiring defibrillation and cardiopulmonary resuscitation (CPR). During CPR, arterial blood gas analysis revealed severe hyponatremia (sodium: 105 mmol/L), while point-of-care ultrasound demonstrated right ventricular distension and bilateral lung-field B-lines. ROSC was achieved after about 13 minutes of CPR. Additional findings included hypokalemia, metabolic acidosis, and elevated lactate levels. The patient was managed with mechanical ventilation, diuretics for pulmonary edema, and vasopressor support before transfer to the intensive care unit after completing surgery. This case underscores the importance of maintaining a high index of suspicion for fluid overload, glycine toxicity, and electrolyte disturbances during hysteroscopic procedures. Rapid deterioration can occur, and point-of-care evaluation during resuscitation may facilitate timely diagnosis and life-saving intervention.

## Introduction

Hysteroscopic myomectomy is a minimally invasive procedure regarded as safe, with low morbidity rates [1,2]. It provides considerable advantages for those experiencing abnormal uterine bleeding from submucosal fibroids, such as fertility preservation, quick recovery, and reduced hospital stay. Recently, hysteroscopic surgeries have become prevalent, with complication rates reported to be as low as 0.24% [3]. However, the figures can increase up to 10% for complicated procedures, such as hysteroscopic myomectomy [4]. The usage of distension media, especially using hypotonic solutions, such as 1.5% glycine, may have hazardous systemic absorption causing dilutional hyponatremia, hypo-osmolality, volume overload, and, rarely, complications that may be fatal [4,5]. In clinical practice, regional anesthesia is preferred because there is no airway instrumentation with all the risks of complications involved. However, intraoperative hemodynamic collapse under regional anesthesia presents a challenge that necessitates prompt airway management and advanced resuscitation.

We present the case of a 36-year-old woman who experienced sudden cardiovascular collapse, electrolyte imbalance, and two episodes of cardiac arrest during hysteroscopic myomectomy. The case underscores the necessity of high vigilance, timely identification of operative hysteroscopic intravascular absorption syndrome (OHIAS), and the importance of intraoperative collaboration and action in averting mortality.

## Case presentation

**Figure 1:**
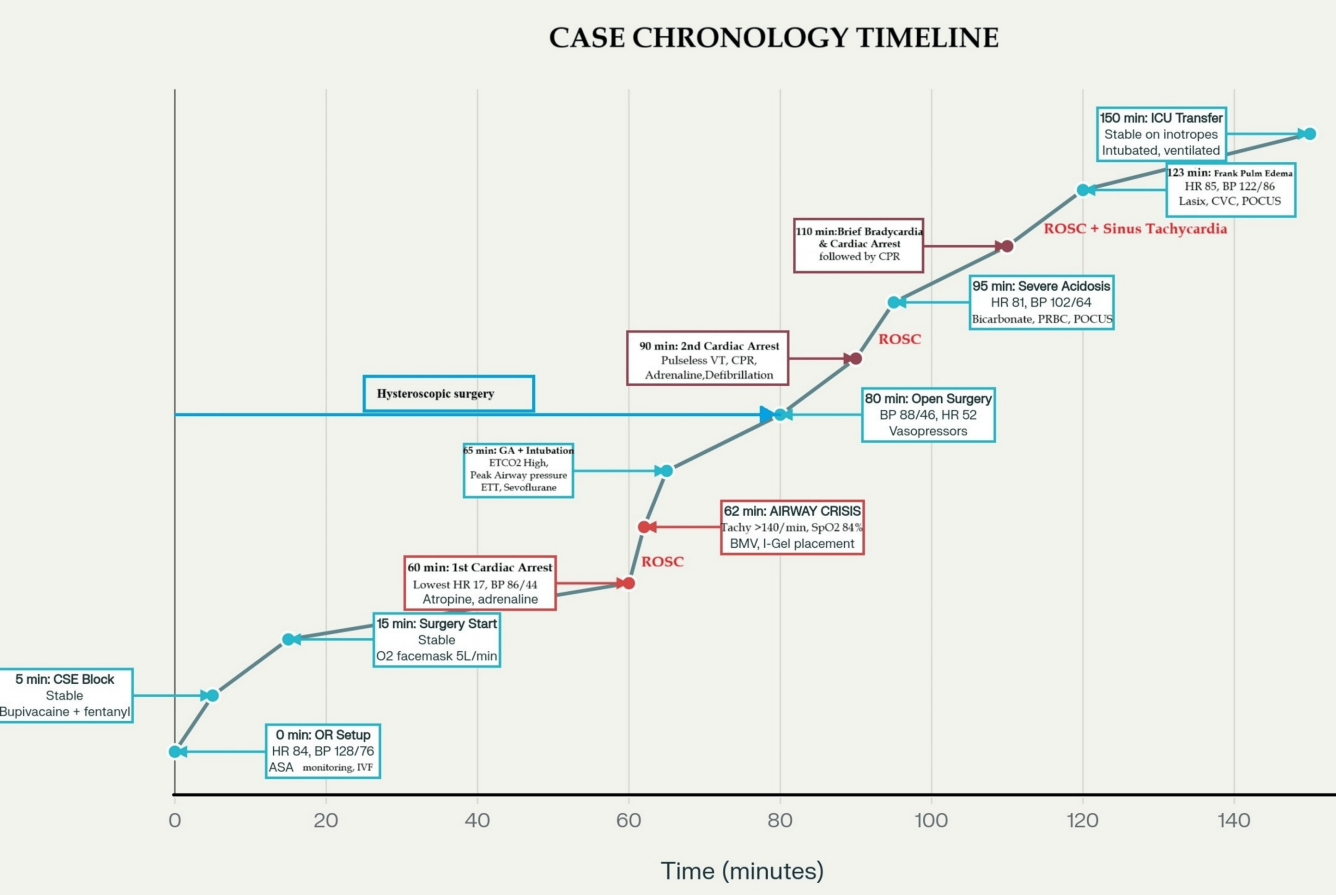
Case timeline and events. ASA: American Society of Anesthesiologists; BMV: bag mask ventilation; BP: blood pressure; CPR: cardiopulmonary resuscitation; CSE: combined spinal epidural; CVC: central venous catheter; EtCO_2_: end-tidal carbon dioxide; HR: heart rate; ICU: intensive care unit; IVF: intravenous fluid; OR: operating room; POCUS: point-of-care ultrasound; PRBC: packed red blood cells; ROSC: return of spontaneous circulation; Tachy: tachycardia; VT: ventricular tachycardia

A 36-year-old woman with a body mass index of 27.5 kg/m², American Society of Anesthesiologists (ASA) physical status I [6], presented with heavy menstrual bleeding for one year. She was diagnosed with a class 2 myoma as per the International Federation of Obstetrics and Gynaecology (FIGO) classification, measuring 42 × 32 × 38 mm, a lobulated submucosal myoma located in the fundal region extending anteriorly, and another FIGO type 5 measuring 12 mm located in the posterior wall. The patient was planned for hysteroscopic myomectomy [7]. She had undergone an uneventful cesarean section seven years ago under spinal anesthesia. Airway examination revealed mild neck movement restriction and modified Mallampati grade IV [8]. The patient’s complete blood parameters were within normal limits. With informed consent and under ASA standards of care monitoring, peripheral venous access with a 20-G cannula was secured. Ringer’s lactate infusion was started, and combined spinal-epidural anesthesia was performed in the sitting position. Bupivacaine 0.5% heavy 2.5 mL, along with 25 μg fentanyl, was administered for a subarachnoid block. A T6 level block was achieved, and the patient was positioned in the lithotomy position. The anesthesia was uneventful until 50 minutes into the procedure, when she developed sudden bradycardia, hypotension, and unresponsiveness, which responded to adrenaline and brief chest compressions (<1 minute) (Annexure 1A). I-gel 4 was used to secure the airway. Return of spontaneous circulation (ROSC) was noted, but elevated airway pressures complicated the case. Therefore, causes were pondered, and, ultimately, the patient’s trachea was intubated under general anesthesia. The hysteroscopic procedure was continued for another 20 minutes, considering near-completion, but required conversion to an open approach. Meanwhile, desaturation was noted, reverted with mechanical ventilation titration, reappeared, and the patient had pulseless ventricular tachycardia requiring defibrillation and cardiopulmonary resuscitation (CPR) (Annexure 1B). During CPR, arterial blood gas analysis revealed severe hyponatremia (sodium: 105 mmol/L; normal: 135-145 mmol/L), while point-of-care ultrasound demonstrated right ventricular distension and bilateral lung-field B-lines. ROSC was achieved after about 13 minutes of CPR (Annexure 1C). Additional findings included hypokalemia, metabolic acidosis, and elevated lactate levels. A chronological timeline of events, vitals, and interventions is presented in Figure 1.

**Figure 2:**
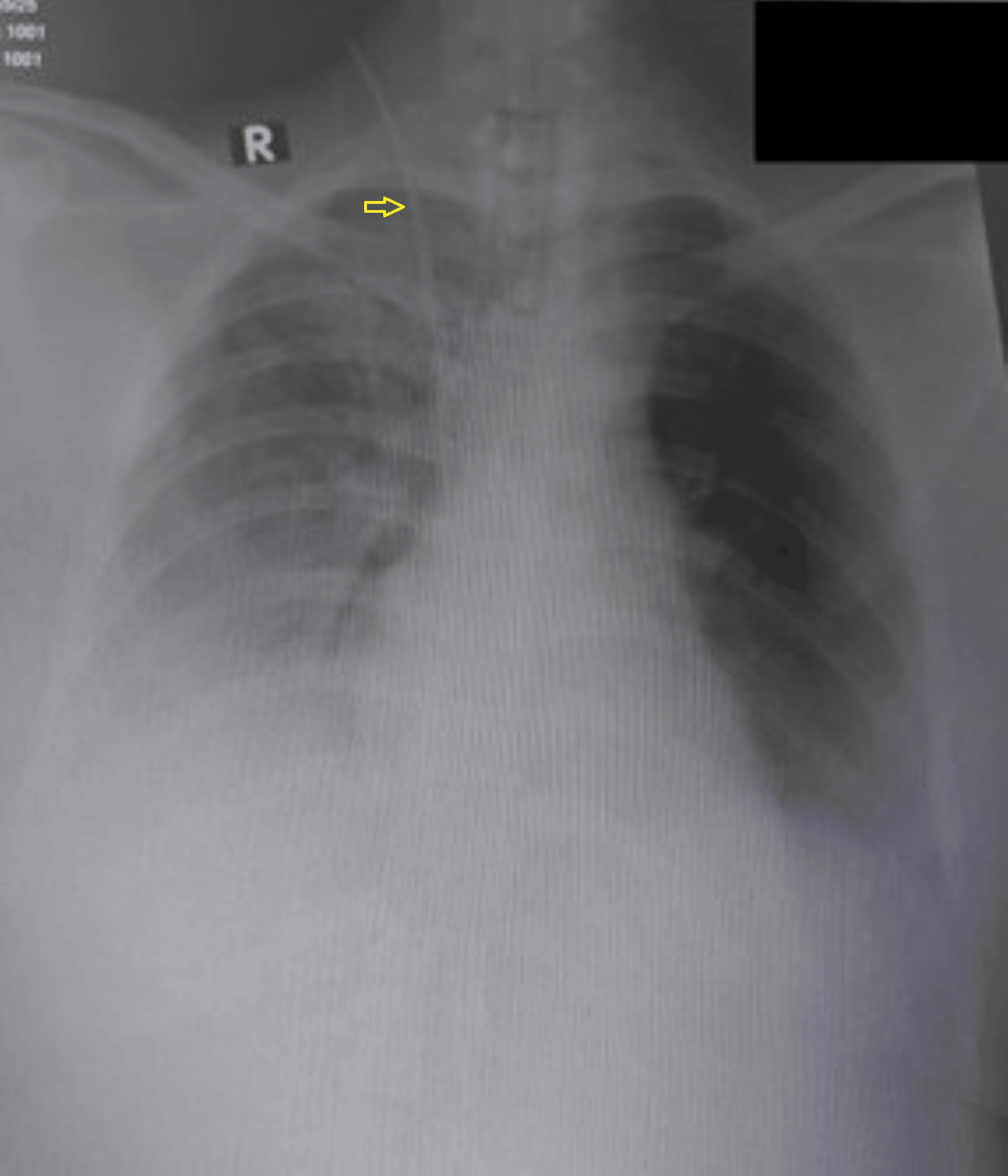
Chest X-ray (anteroposterior view) showing bilateral diffuse haziness, more prominent in the lower zones, with blurred lung markings. The right internal jugular vein central catheter is in situ (arrow).

In the intensive care unit (ICU), the ECG showed a normal sinus rhythm. The inferior vena cava diameter was 1.9 cm, with no respiratory variation noted. The two-dimensional echocardiogram indicated global hypokinesia of the left ventricle, with an ejection fraction of 30%, and dilatation. No regional wall motion abnormalities, mitral regurgitation, aortic regurgitation, with a pulmonary artery acceleration time of 120 ms, or pericardial effusion was observed. Tricuspid annular plane systolic excursion was 15 mm. The lung exhibited a B profile. The diameter of the optic nerve sheath was 3.3 mm on the right and 3.5 mm on the left side, with no signs of increased intracranial pressure. The chest X-ray (also suggested pulmonary edema (Figure 2).

The patient was managed conservatively with mechanical ventilation support, injection furosemide 40 mg, and intravenous antibiotics. A bolus of 100 mL 3% sodium chloride was also administered over 10 minutes, and neuromuscular blockade was reversed. Cardiac injury markers, electrolytes, liver enzymes, and complete blood counts are presented in Table 1. Epidural infusion was continued with isobaric ropivacaine at 0.15% and fentanyl at 2 μg/mL, administered at 5 mL/hour. The 24-hour records of fluid input and output showed 2.3 L and 6.1 L, respectively, with a negative fluid balance of 3.8 L. On fentanyl infusion at 30 μg/hour, the patient was kept sedated and extubated on the next day after attaining full consciousness and the capacity to follow verbal commands. After extubation, the patient was kept on a nasal cannula, where she received oxygen at a flow rate of 2 L/minute. Arterial blood gas analysis showed near-normal results (Table 1).

**Table 1:** Laboratory test results and their reference values. ALT: alanine aminotransferase; AST: aspartate aminotransferase; NT-proBNP: N-terminal pro-B-type natriuretic peptide; ROSC: return of spontaneous circulation; HCO_3_: bicarbonate; PCO_2_: partial pressure of carbon dioxide; PO_2_: partial pressure of oxygen

Parameter	Value reported	Biological reference values
Cardiac biomarkers – 2 hours after ROSC		
Troponin I	0.084 ng/mL	<0.1 ng/mL
NT-proBNP	89.9 pg/mL	<100 pg/mL
Complete blood count – postoperative day 1, morning		
Hemoglobin	11.9 g/dL	Female: 12 – 16 g/dL
Total leukocyte count	22,310 cells/μL	4,000–11,000 cells/μL
Platelet count	140,000 cells/μL	150,000–450,000 cells/μL
Sodium (serum)	133 mEq/L	135–145 mEq/L
Potassium (serum)	4.3 mEq/L	3.5–5.0 mEq/L
Chloride	104 mEq/L	98–106 mEq/L
Electrolytes – postoperative day 1, evening		
Sodium (serum)	137 mEq/L	135–145 mEq/L
Potassium (serum)	3.2 mEq/L	3.5–5.0 mEq/L
Chloride	101 mEq/L	98–106 mEq/L
Liver functions – postoperative day 1, morning		
Albumin	2.9 g/dL	3.5–5.5 g/dL
ALT	359 IU/L	7–56 IU/L
AST	299 IU/L	10–40 IU/L
Arterial blood gas – post-extubation		
PCO_2_	37 mmHg	35–45 mmHg
PO_2 _	77 mmHg	80–100 mmHg (room air)
HCO_3_	24 mmol/L	22–26 mmol/L
pH	7.44	7.35–7.45
Hemoglobin	8.5 g/dL	12–16 g/dL
Lactate	1.18 mmol/L	0.5–2.2 mmol/L

Subsequently, the lung ultrasound showed an A-profile, while the chest X-ray revealed clear lung fields. The patient was transferred out of the ICU on the third postoperative day and discharged from the hospital on the seventh postoperative day.

## Discussion

The present case highlights a sudden intraoperative cardiorespiratory collapse caused by excessive systemic absorption of distension fluid, severe acute hyponatremia during hysteroscopic myomectomy (transurethral resection of the prostate (TURP)-like syndrome or OHIAS), myocardial stunning from prolonged hypoxia and electrolyte disturbance and arrest, and subsequent full recovery with rapid sodium correction, negative fluid balance, and artificial respiratory support. This case emphasizes the importance of close hemodynamic and fluid balance monitoring and a prompt multidimensional approach, including point-of-care diagnostics, during hysteroscopic procedures.

Although the hysteroscopic removal of symptomatic submucosal fibroids is minimally invasive, fatal hemodynamic collapse, including arrest, might result from OHIAS, also referred to as a TURP-like syndrome [9,10]. During hysteroscopy, non-electrolyte hypotonic solutions, such as 1.5% glycine, are frequently used because they can be used with monopolar electrosurgery. When intrauterine pressure exceeds that present in the venous circulation, open venous sinuses bulge into the myometrium, and the irrigated fluid may be absorbed into the systemic circulation. Excessive absorption results in dilutional hyponatremia and hypo-osmolality, subsequently causing cerebral edema, seizures, confusion, and coma [11]. Hypokalemia occurs from intracellular shifts in potassium, predisposing patients to arrhythmias. Further, metabolism of glycine into ammonia and glyoxylic acid can potentially contribute to neurotoxicity. Volume overload precipitates pulmonary edema, hypoxemia, and congestive cardiac failure [12,13].

Restlessness, nausea, vomiting, blurred vision, and bradycardia are early signs. Further symptoms such as hypotension, arrhythmias, hypoxemia, seizures, or cardiac arrest may develop as absorption continues [13,14]. Our patient’s critical hyponatremia, hypokalemia, acidosis, and pulmonary edema were consistent with OHIAS.

Intraoperative collapse during hysteroscopy may arise from reflex cardiovascular depression (also termed as vasovagal syncope, the Bezold-Jarisch reflex, and neurocardiogenic syncope), anaphylaxis, venous air embolism, high spinal or total spinal, severe dyselectrolytemia, and glycine toxicity [15,16]. In our case, the absence of a rash, bronchospasm, tongue swelling, and abrupt pulmonary edema after an hour ruled out anaphylaxis. Moreover, the absence of “mill-wheel murmur” or sudden desaturation and no air in focused echocardiography in the right heart chamber ruled out venous air embolism. The incident occurred suddenly after an hour of spinal anesthesia, and the patient had no weakness or tingling in the hand until the time before collapse ruled out the possibility of high or total spinal anesthesia. Interestingly, the patient also did not show any progressive signs or symptoms of glycine toxicity. Further, electrolyte imbalance is integral to OHIAS, making it the most consistent diagnosis. Nevertheless, we also suspected reflex cardiovascular collapse at the beginning, as volume overload-related features were not apparent, and the cervical stimulation was coincident with the abrupt collapse. The management of OHIAS involves the following critical steps: (1) immediate cessation of surgery to prevent further absorption of the distension fluid, (2) aggressive resuscitation and airway management, (3) correction of electrolytes and acid-base disorders, and (4) advanced life support for a cardiac arrest patient. Two episodes of ventricular tachycardia required CPR, defibrillation, and amiodarone.

Regional anesthesia was preferred, considering pelvic surgery was anticipated to be a difficult airway. However, airway control may become necessary during regional anesthesia when systemic complications occur. The patient’s trachea was intubated by an experienced person using the C-MAC system. Vigilance of intraoperative fluid administration is crucial. The absorption threshold for hypotonic solutions such as glycine is 1,000 mL, above which the risk of OHIAS increases [17]. The irrigation fluid deficit was not recorded due to technical issues, delaying syndrome recognition. Multiple factors contribute to absorption, including prolonged operation (>45-60 minutes), large myomas with wide venous sinuses, mean blood pressure <65 mmHg, high intrauterine pressure (>80 mmHg), and hypotonic non-electrolyte fluids [18]. Our patient had all these risk factors, i.e., procedure time >60 minutes, uncertain intrauterine pressure, and the use of 1.5% glycine solution. The fluid management should thus be meticulous; irrigation fluid in and out should be recorded constantly, and maximum fluid deficit thresholds should be strictly adhered to (1,000 mL for hypotonic solutions and 2,500 mL for isotonic saline) [17,18]. For older adults or comorbid patients, a lower threshold of 750 mL for hypotonic solutions and 1,500 mL for isotonic solutions is advocated [17]. Further, intraoperative mean arterial pressure should be maintained above 65 mmHg, preferably on the higher side, throughout the procedure. Bipolar resectoscopes can also use normal saline 0.9% as an alternative distention medium, which eliminates the risk of hyponatremia. Early recognition and intervention in cases of hysteroscopy with intraoperative bradycardia, hypotension, or unexplained neurological signs should heighten the suspicion of possible OHIAS. Preparedness for airway management, even under regional anesthesia, is crucial in emergencies. Perioperative survival depended on multidisciplinary teamwork, closed-loop communication, and coordination between the surgical, anesthesia, and ICU teams [19]. Acute symptomatic hyponatremia with serum Na^+^ level <120 meq/L should be treated with 3% normal saline 100 mL bolus over 10 minutes, and, if required, can be repeated up to three times [20]. Although the British Society for Gynaecological Endoscopy/European Society for Gynaecological Endoscopy Guideline Development Group for Management of Fluid Distension Media in Operative Hysteroscopy does not specifically mention the duration, the increasing duration of hysteroscopy increases the chance of OHIAS [20].

## Conclusions

OHIAS is a catastrophic complication in a routine, minimally invasive, hysteroscopic surgery procedure. It demands prompt recognition, differentiation using point-of-care evaluations, and team dynamics for managing such events. Sudden hemodynamic collapse in hysteroscopic surgery should raise concerns, and diligent and frequent fluid balance monitoring is crucial. Our case underscores that calling for immediate help, emergent airway management, initiation of timely resuscitation, CPR, and searching for possible reversible causes of arrest is expected to result in a favorable outcome.

## Figures and Tables

**Figure 3:**
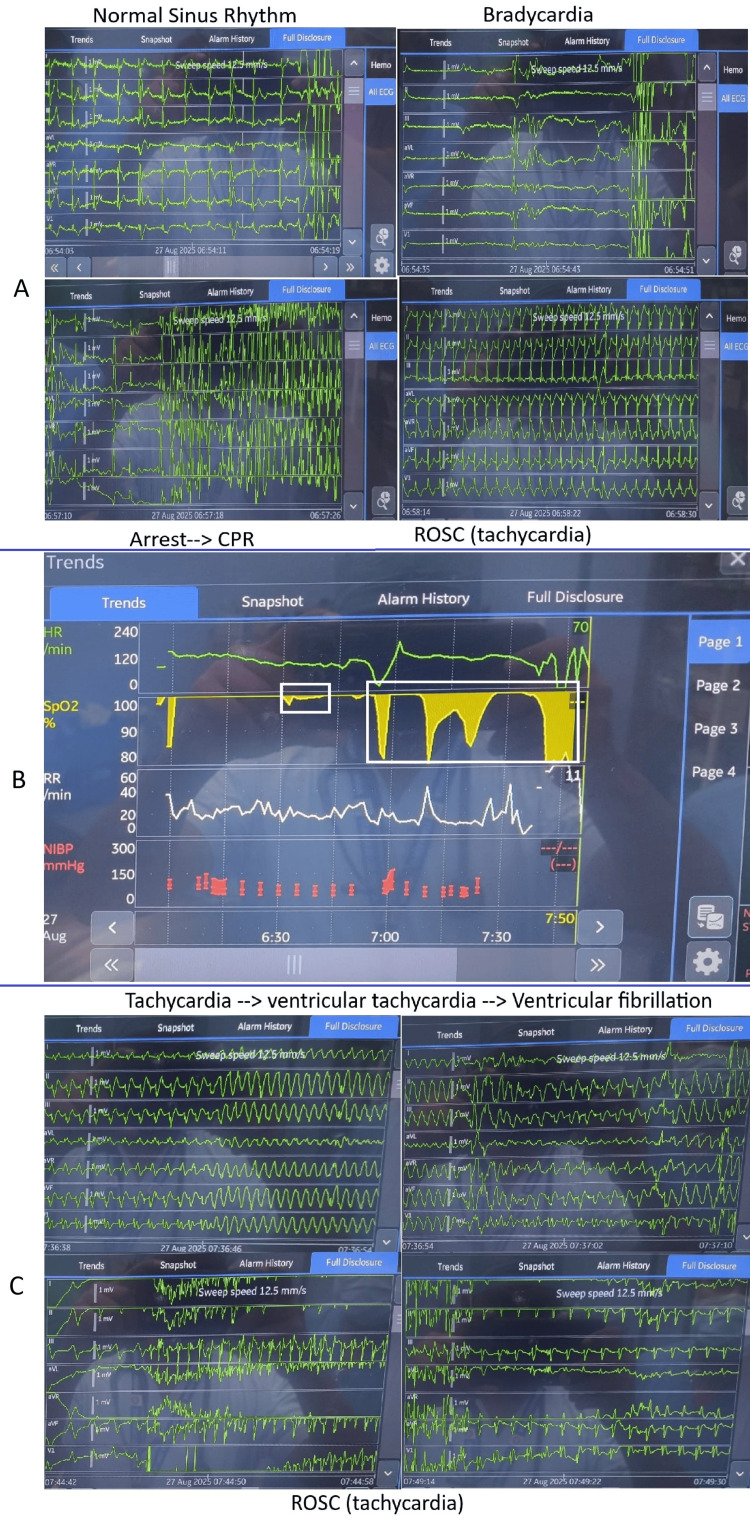
Trend disclosure from the anesthesia monitor. (A) Brief bradycardia followed by arrest and ROSC. (B) Disclosure of saturation trend where the rectangles are used to show the desaturation episodes. (C) Ventricular tachycardia, arrest, tachycardia after ROSC. CPR: cardiopulmonary resuscitation; ROSC: return of spontaneous circulation
